# Clinical observation and mechanism of acupuncture on amnestic mild cognitive impairment based on the gut-brain axis: study protocol for a randomized controlled trial

**DOI:** 10.3389/fmed.2023.1198579

**Published:** 2023-06-21

**Authors:** Qiongnan Bao, Yiwei Liu, Xinyue Zhang, Yaqin Li, Ziqi Wang, Fang Ye, Xia He, Manze Xia, Zhenghong Chen, Jin Yao, Wanqi Zhong, Kexin Wu, Ziwen Wang, Mingsheng Sun, Jiao Chen, Xiaojuan Hong, Ling Zhao, Zihan Yin, Fanrong Liang

**Affiliations:** ^1^School of Acu-Mox and Tuina, Chengdu University of Traditional Chinese Medicine, Chengdu, China; ^2^Sichuan Provincial Acupuncture Clinical Medicine Research Center, Chengdu, China; ^3^The West China Hospital, Chengdu, China; ^4^The Fourth People's Hospital of Chengdu, Chengdu, China; ^5^The Sichuan Province People's Hospital, Chengdu, China; ^6^The Rehabilitation Hospital of Sichuan Province, Chengdu, China

**Keywords:** acupuncture, amnestic mild cognitive impairment, randomized controlled trial, gut-brain axis, protocol

## Abstract

**Background:**

Amnestic mild cognitive impairment (aMCI) is a pre-dementia condition associated with declined cognitive function dominated by memory impairment. The occurrence of aMCI is associated with the gut-brain axis. Previous studies have shown cognitive improvements in MCI after acupuncture treatment. This study evaluates whether acupuncture can produce a therapeutic effect in patients with aMCI by modulating the gut-brain axis.

**Methods and design:**

This is a prospective, parallel, multicenter randomized controlled trial. A total of 40 patients with aMCI will be randomly assigned to an acupuncture group (AG) or a waiting-list group (WG), participants in both groups will receive health education on improving cognitive function at each visit, and acupuncture will be conducted twice a week for 12 weeks in the AG. Another 20 matched healthy volunteers will be enrolled as normal control. The primary outcome will be the change in Alzheimer’s Disease Assessment Scale-cognitive scale score before and after treatment. Additionally, functional magnetic resonance imaging data, faeces, and blood will be collected from each participant to characterize the brain function, gut microbiota, and inflammatory cytokines, respectively. The differences between patients with aMCI and healthy participants, and the changes in the AG and WG groups before and after treatment will be observed. Ultimately, the correlation among brain function, gut microbiota, inflammatory cytokines, and clinical efficacy evaluation in patients with aMCI will be analyzed.

**Discussion:**

This study will identify the efficacy and provide preliminary data on the possible mechanism of acupuncture in treating aMCI. Furthermore, it will also identify biomarkers of the gut microbiota, inflammatory cytokines, and brain function correlated with therapeutic effects. The results of this study will be published in peer-reviewed journals.

**Clinical trial registration:**

http://www.chictr.org.cn, identifier ChiCTR2200062084.

## 1. Introduction

Mild cognitive impairment (MCI) refers to a transitional phase between cognitive changes of normal ageing and dementia, which is often seen in Alzheimer’s disease (AD) (1). It is characterized by a decline in memory, language and executive function (2). The prevalence of MCI varies between 10% and 20% in adults aged ≥65 years and increases with age (3), and approximately 5 million individuals in the United States aged ≥65 years have MCI (4). The proportion of people with MCI increased from 11.9% in people aged 60–69 years, 19.3% in those aged 70–79 years, and 24.4% in those aged 80–89 years in China (5). The World Health Organization has reported that more than 245 million people in the Western Pacific Region are >65 years old, with this number projected to double by 2050 (6). Thus, the number of patients with MCI tends to increase.

MCI is classified into amnestic MCI (aMCI) and non-amnestic MCI (naMCI) (2). As the most common subtype of MCI, aMCI refers to a syndrome of cognitive decline dominated by memory impairment, with preserved independent functional abilities (7). Individuals with aMCI are more likely to deteriorate to dementia than the age-matched normal population or people with naMCI (8, 9). Because of the progressive decline in cognition, aMCI markedly affects the patient’s quality of life and imposes a substantial financial burden on families and society (10). In the United States, patients with aMCI have significantly less annual household income (average of $29,754) compared to individuals with normal cognition (average of $45,500) (11). This makes aMCI a global concern that needs urgent attention and solutions.

Early recognition and effective intervention for aMCI are critical for slowing down disease progression (4). Based on the updated guideline of the American Academy of Neurology, no pharmacological or dietary agents have been proven to improve the clinical symptoms or delay the progression of aMCI (12). The efficacy of traditional drugs is insufficient (13). For example, cholinesterase inhibitors have limited effects on cognitive function over the short-term but substantially high adverse effects (14). Other management options recommended for aMCI, such as lifestyle changes, aerobic exercise and social engagement, also seem unsatisfactory (3). Therefore, alternative interventions to manage aMCI have become of interest.

As a traditional Chinese medicine (TCM) component, acupuncture has been applied to various neurological diseases for thousands of years. According to TCM theory, acupuncture involves inserting needles into acupoints to adjust Qi and blood of meridians and balance yin and yang throughout the body, thus normalizing the patient’s health status (15). Abundant randomized controlled trials (16, 17) and systematic reviews/meta-analyses (18, 19) have shown that acupuncture can be used to treat MCI. Xu et al. (20) found that the effect of acupuncture on MCI was better than western medicines. Additionally, our previous systematic review (18) indicated that acupuncture is an effective therapy for overall cognitive function improvement in patients with MCI. Acupuncture seems to be a promising complementary therapy for aMCI; however, the underlying mechanisms of acupuncture are still under debate.

Previous studies found that patients with aMCI have changes in brain structure and function, including reduced cortical thickness, and abnormal activity and functional connectivity in specific brain regions (21, 22). In recent years, evidence has established that aMCI occurrence is associated with gut microbiota alteration and neuroinflammatory response (23). Changes in gut microbiota and its metabolites interact with human brain development and cognitive function (24, 25). The dynamic bidirectional connection pathway between the gastrointestinal tract and the nervous system is termed the “gut-brain axis” (26). Gut microbiota taxa could be used as key early indicators of cognitive performance in patients with aMCI (23). Sodium oligomannate has been demonstrated to reverse cognitive impairment by remodelling the gut microbiome and limiting neuroinflammation (27). Based on this, gut microbiome dysbiosis and neuroinflammation may be critical factors contributing to cognitive impairment in patients with aMCI. In recent years, researchers found that acupuncture improved cognition in animal models of AD by modulating the microbiota-gut-brain axis (28–30). Additionally, a review demonstrated that acupuncture exerts an anti-neuroinflammation effect through multiple pathways (31). Thus, we hypothesise that acupuncture may improve the cognitive function of patients with aMCI by regulating the gut-brain axis.

Evidently, the gut-brain axis may play a crucial role in the aMCI pathogenesis. To our knowledge, no study has been conducted to elucidate the underlying mechanisms of acupuncture for aMCI based on the gut-brain axis. Therefore, we designed a parallel-arm, randomised, controlled clinical trial to explore the therapeutic mechanisms of acupuncture for aMCI from the perspective of gut-brain modulation.

## 2. Objectives

1. To observe the efficacy and safety of acupuncture in patients with aMCI;
2. To investigate whether acupuncture can regulate brain function by affecting the gut microbiome and expression of inflammatory cytokines in the gut-brain axis, thus playing a role in improving cognitive function in patients with aMCI.

## 3. Design and methods

### 3.1. Trial design and setting

**Figure 1:**
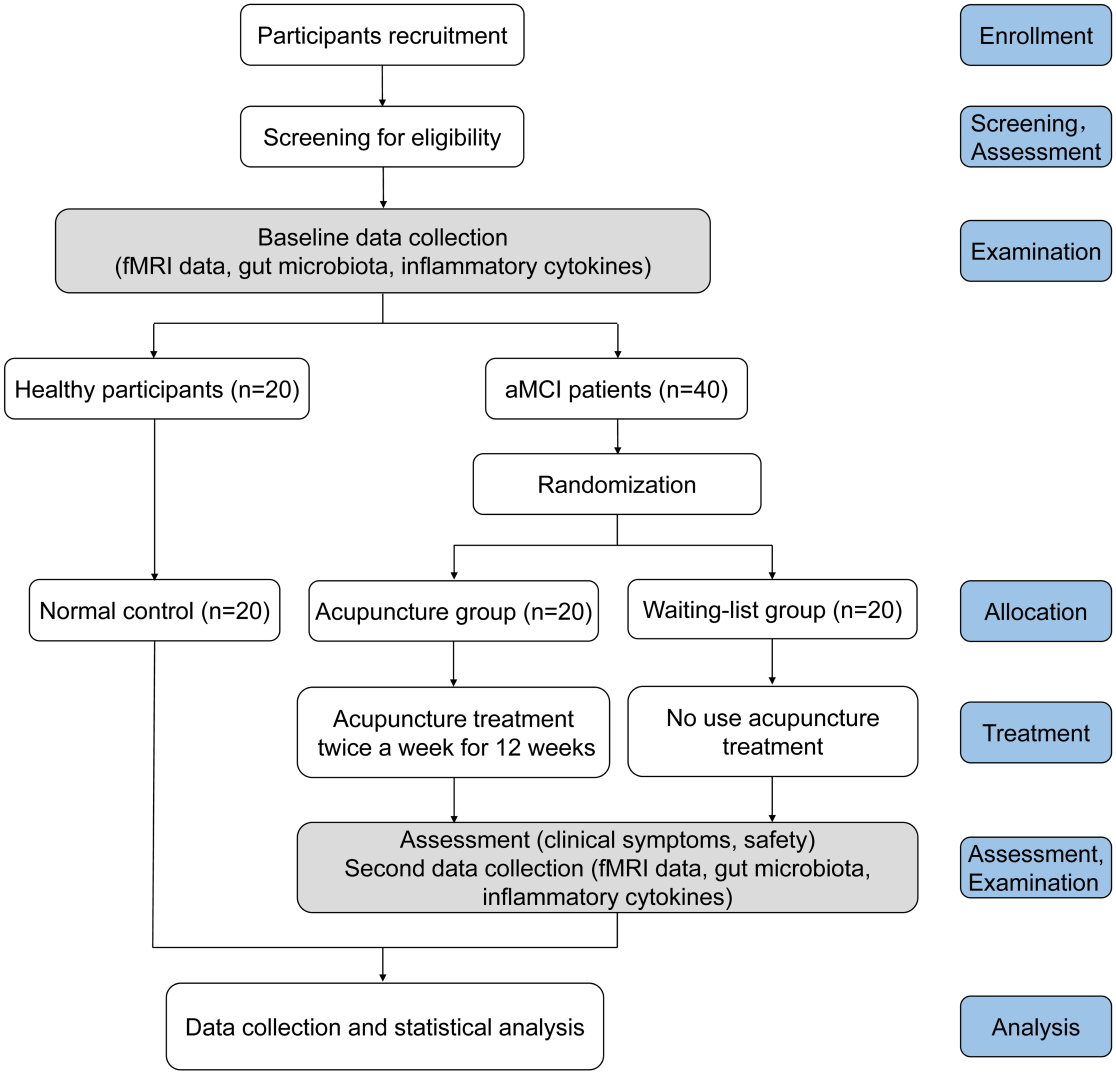
Flow chart of the trial procedures.

This is a parallel-designed, prospective, assessor-blinded multicentre (*n* = 4) randomised controlled study to explore whether acupuncture can improve clinical symptoms of patients with aMCI by regulating the gut-brain axis. Forty patients with aMCI will be randomly and evenly assigned to either the acupuncture group (AG) or the waiting-list group (WG). Various scales will be assessed, functional Magnetic Resonance Imaging (fMRI) scans will be performed, and blood and faeces samples will be collected from both groups of patients before and after the intervention. In addition, 20 healthy participants will be recruited as normal control, and relevant tests and examinations will be conducted at enrollment. Cognitive-related scale, daily-living ability scale, emotion scale, mental behaviour scale and sleep scale will be used to observe the changes in clinical symptoms of patients with aMCI and to identify the therapeutic effect of acupuncture on aMCI. The gut-brain interaction characteristics of patients will be determined, and the regulatory effect of acupuncture on gut microbiota, inflammation cytokines and brain function will be explored. Subsequently, the correlation analysis of clinical efficacy can be related to gut microbiota composition, inflammation cytokines and brain function. The flow chart of trial procedures is displayed in Figure 1, and the study schedule is depicted in Table 1. The trial will be conducted at four sub-centres (West China Hospital of Sichuan University, Sichuan Provincial People’s Hospital, Chengdu Fourth People’s Hospital, and Sichuan Provincial Rehabilitation Hospital). The study protocol reporting follows the Standard Protocol Items: Recommendations for Interventional Trials (SPIRIT) guidelines and checklist (32).

**Table 1:** Schedule of enrollment, intervention, and assessments.

Timepoint	Study period				
	aMCI patients				Healthy participants
	Baseline	Treatment phase			Baseline
	0-week	Week 1	→	Week 12	**0-week**
Enrollment:					
Eligible screen	X				X
Informed consent	X				X
Randomization	X				
Interventions:					
Acupunctue group		X	→	X	
Waiting-list group		X	→	X	
Examinations:					
fMRI scan	X			X	X
Blood detection	X			X	X
Feces detection	X			X	X
Assessments:					
ADAS-cog	X			X	X
AVLT-H	X			X	X
MoCA-B	X			X	X
FAQ	X			X	X
HAMD	X			X	X
HAMA	X			X	X
MBI-C	X			X	X
PSQI	X			X	X
Adverse events		X	→	X	

aMCI, amnestic mild cognitive impairment; ADAS-cog, alzheimer’s disease assessment scale-cognitive subscale; AVLT-H, auditory verbal learning test – huashan; MoCA-B, montreal cognitive assessment - basic; FAQ, functional activities questionnaire; HAMA, hamilton anxiety scale; HAMD, hamilton depression scale; MBI-C, mild behavioral impairment-checklist; PSQI, pittsburgh sleep quality index; fMRI, functional magnetic resonance imaging.

### 3.2. Participants

Eligible participants will be recruited from clinical centres, communities and welfare institutes in Sichuan province. Written informed consent will be obtained before participation if the participants meet the inclusion criteria.

### 3.3. Eligibility criteria

#### 3.3.1. Inclusion criteria for patients with aMCI

Patients who meet the following criteria will be included: (1) diagnosis of aMCI according to the Jak/Bondi 2014 diagnostic criteria (33); (2) right-handedness and age between 50 and 80 years; (3) disease course ≥6 months; (4) a Clinical Dementia Rating (CDR) score of 0.5; (5) a Hachinski Incheinic Score (HIS) ≤ 4; (6) education level ≥ 8 years (including vocational education); (7) volunteering to cooperate and signing an informed consent form; and (8) no contraindications to MRI scanning, such as pacemaker implantation, metal fixed dentures.

#### 3.3.2. Exclusion criteria for patients with aMCI

Patients who meet any of the following criteria will be excluded: (1) receiving treatment that interferes with cognitive function (e.g., management of acute psychotic episodes, such as memantine, rivasmine, donepezil); (2) a history of neurological conditions affecting cognitive function confirmed by examination, except in patients with suspected early AD, including Parkinson’s disease, vascular dementia, brain tumour, traumatic brain injury, or other diseases which might lead to neurological injury and abnormal brain structure; (3) presence of a systemic diseases that could cause cognitive decline, such as anaemia, Hashimoto’s encephalopathy, metabolic encephalopathy, hepatic encephalopathy, renal encephalopathy; (4) infection, other focal injury, multiple or important brain memory area infarcts, or severe leukodystrophy (Fazekas score ≥ 3) indicated by brain MRI; (5) a history of tumour, psychiatric illness (e.g., bipolar disorder, schizophrenia) or severe depression (Hamilton Depression [HAMD] scale score ≥ 24) and anxiety (Hamilton anxiety [HAMA] scale score ≥ 29); (6) haemorrhagic disease, bleeding tendency or severe skin infection; (7) severe drug dependence, smoking, drug or alcohol abuse; (8) pregnant, potentially pregnant, or lactating females; (9) receipt of any acupuncture therapy or participation in other clinical trials during the 6 months prior to enrolment; and (10) regularly use of probiotics, prebiotics, or antibiotics.

#### 3.3.3. Inclusion criteria for healthy participants

Participants who meet all of the following criteria will be included as normal control: (1) right-handedness and age ≥ 50 years and < 80 years; (2) no cognitive impairment according to Jak/Bondi criteria (non-subjective cognitive decline/MCI/dementia) and normal daily living ability (a Functional Activities Questionnaire [FAQ] score < 9); (3) no contraindications to MRI scanning, such as pacemaker implantation, metal fixed dentures; and (4) volunteering to cooperate and signing an informed consent form.

#### 3.3.4. Exclusion criteria for healthy participants

Participants who meet any of the following criteria will be excluded from the normal control group: (1) claustrophobia; (2) severe cranial anatomical asymmetry or definite lesions found in MRI; (3) regularly use of probiotics, prebiotics, or antibiotics; or (4) participation in other clinical trials.

### 3.4. Randomization and assessor-blinding

Patients with aMCI will be randomly divided into AG or WG according to the computer-generated random numbers. The random sequence with an identifying letter will be sealed in a light-tight envelope, which will be opened when eligible participants are enrolled. During the research process, the sequence generation and allocation will be performed by an independent person not participating in the trial. Healthy participants will be enrolled as the normal control without randomization.

Blinded evaluation will be conducted, and patients will be treated separately. The efficacy will be evaluated by a third person unaware of the group assignments; blinded statistical analysis will be used in the data summary stage, and the investigators, clinical operators, efficacy evaluators, and data statisticians will be separated throughout the whole study process.

### 3.5. Sample size

Previous studies (34, 35) have suggested that 15 participants should be included in each group to ensure a stable statistical effect for brain fMRI data analysis. In most similar studies (36–38) which used fMRI, gut microbiota, or inflammatory cytokines as indicators to explore the mechanisms of acupuncture, the sample size was mostly 15–20 participants per group. Thus, the sample size in our study will be 20 participants per group. Additionally, 20 healthy participants will be included.

### 3.6. Intervention

#### 3.6.1. Patients with aMCI

**Figure 2:**
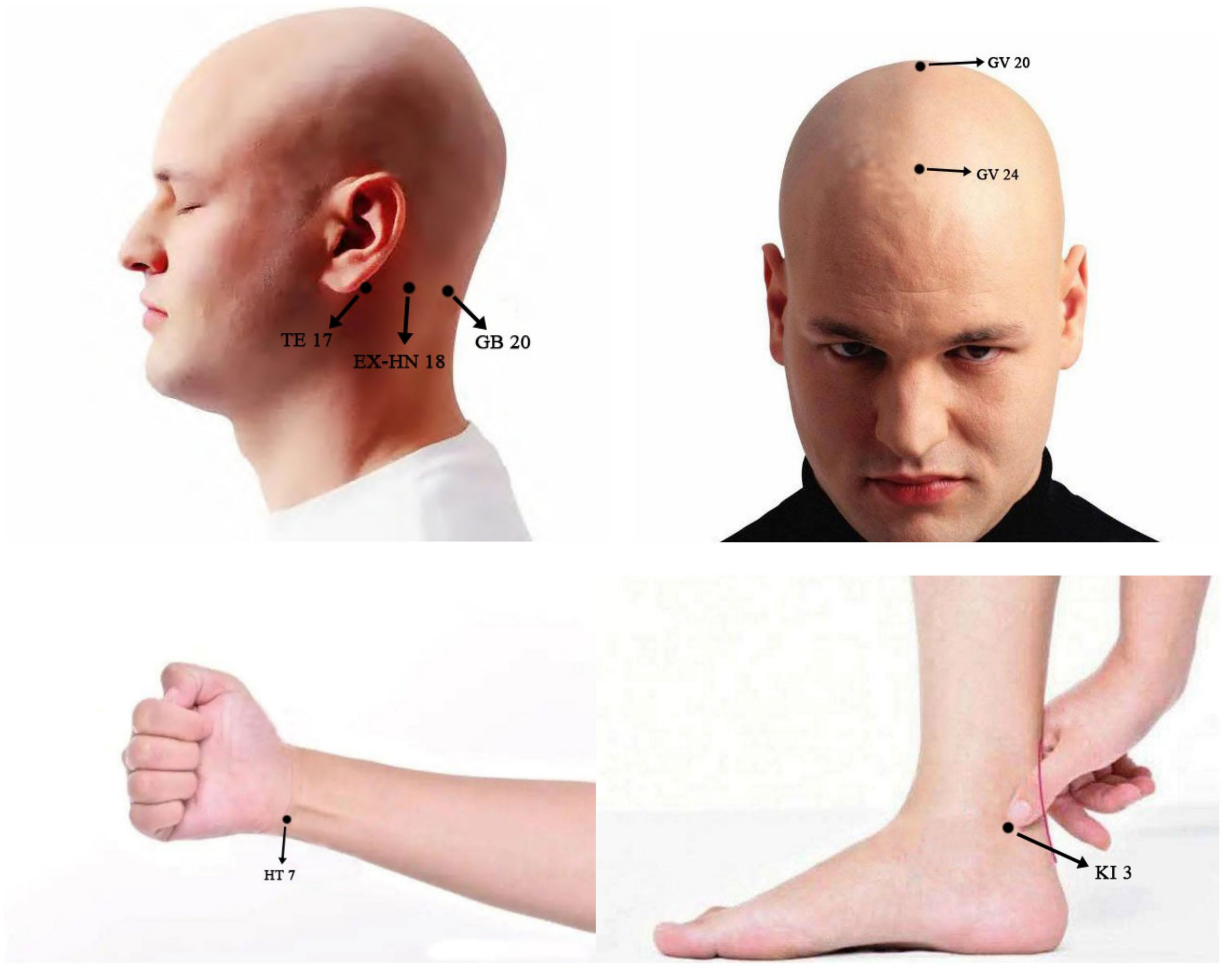
Locations and manipulations of acupoints selected in this study: GV 20 (Baihui), 5 cun directly above the midpoint of the anterior hairline, at the midpoit of the connecting the apexes of the two auricles. Subcutaneous insertion to a depth of 0.5-1 cun with manipulation for the deqi. GV 24 (Shenting), 0.5 cun directly above the midpoint of the anterior hairline. Subcutaneous insertion to a depth of 0.3-0.5 cun with manipulation for the deqi. EX-HN 18 (Anmian), at the neck, the midpoint of the line between GB20 (Fengchi) and TE 17 (Yifeng). Subcutaneous insertion to a depth of 0.5-1 cun with manipulation for the deqi. HT 7 (Shenmen), at the ulnar end of the transverse crease of the wrist, in the depression on the radial side of the tendon of m. flexor carpi ulnaris. Subcutaneous insertion to a depth of 0.3-0.5 cun with manipulation for the deqi. KI 3 (Taixi), posterior to the medial malleolus, in the depression between tip of the medial malleolus and tendo calcaneus. Subcutaneous insertion to depth of 0.5-1 cun with manipulation for the deqi.

Patients in the AG will receive acupuncture treatment. Standardised acupuncture treatment will be based on the Standards for Reporting Interventions in Controlled Trials (STRICTA) (Table 2). The following acupoints will be used: Shenting (GV 24), Baihui (GV 20), Anmian (EX-HN 18), Shenmen (HT 7), and Taixi (KI 3). The orientation and manipulation of the acupoints will be performed based on TCM standards, as described in Figure 2. Licensed acupuncturists will use single-use sterile needles (Hwato, Suzhou, China; 0.25 × 25 mm) to insert acupoints after skin disinfection. Subsequently, twisting, thrusting, and rotation will be conducted by uniform reinforcing-reducing methods to create the experience termed De Qi within the range of patient tolerance. All needles will be manipulated manually every 10 min to maintain the De Qi sensation, the twisting angle will be 90–180 degrees, and the frequency will be 60–90 times/min, with an amplitude of lifting and inserting of 0.3–0.5 cm. The treatment will include 24–30 min sessions over 12 weeks.

**Table 2:** Acupuncture treatment details based on the STRICTA 2010 checklist.

Item	Item number	Detail
1. Acupuncture rationale	1a) Style of acupuncture	Traditional Chinese Medicine
	1b) Reasoning for treatment provided, based on historical context, literature sources, and/or consensus methods, with references where appropriate	The treatment is carried out according to traditional acupuncture theory, previous studies, and the experts’ consensus.
	1c) Extent to which treatment was varied	Standardised acupuncture treatment
2. Details of needling	2a) Number of needle insertions per subject per session	8
	2b) Names of points used	GV 20 (Baihui), GV 24 (Shenting), EX-HN 18 (Anmian), HT 7 (Shenmen), and KI 3 (Taixi)
	2c) Depth of insertion, based on a specified unit of measurement, or on a particular tissue level	From 15 to 25 mm.
	2d) Response sought	Deqi (numbness, soreness, heaviness, distention, etc.)
	2e) Needle stimulation	Manual acupuncture
	2f) Needle retention time	30 min
	2g) Needle type	Sterile, disposable acupuncture needles (length, 25 mm; diameter, 0.25 mm; Hwato, China).
3. Treatment regimen	3a) Number of treatment sessions	24 treatment sessions in acupuncture group.
	3b) Frequency and duration of treatment sessions	Twice per week (once per 2–3 days interval), for 12 continuous weeks.
4. Other components of treatment	4a) Details of other interventions administered to the acupuncture group	Dietary and lifestyle advice.
	4b) Setting and context of treatment, including instructions to practitioners, and information and explanations to patients	The trial will be implemented at departments (Chengdu Fourth People’s Hospital, Sichuan Provincial People’s Hospital, West China Hospital of Sichuan University, Sichuan Provincial Rehabilitation Hospital). All participants of acupuncture group will be arranged to the acupuncture & moxibustion room for treatment. All information and explanations will be provided to participants.
5. Practitioner background	5) Description of participating acupuncturists	Trained, licensed acupuncturists with at least 6 years in acupuncture clinical practice
6. Control or comparator interventions	6a) Rationale for the control or comparator in the context of the research question, with sources that justify this choice	The treatment is carried out according to previous studies, and the experts’ consensus.
	6b) Precise description of the control or comparator. If sham acupuncture or any other type of acupuncture-like control is used, provide details as for Items 1 to 3 above.	No acupuncture treatment in the waiting-list group during 12-week observation period after randomization. And dietary and lifestyle advice would be provided at each visit.

Patients in the WG will not undergo acupuncture treatment during the observation period, and they will be informed that 24 free acupuncture sessions can be provided to them after observation.

Both groups will receive 10–15 min of aMCI-related health education at each visit to improve their cognitive function. The health education content is referred to in the *Dietary and lifestyle guidelines for the Prevention of Alzheimer’s Disease,* published in *Neurobiology of Aging* in 2014 (39). During the trial, patients will be allowed to receive basic treatment, such as blood pressure control, glucose control, and other supportive care. The time, dose, frequency, and response to treatment will be recorded. Participants will also be advised to avoid using other medications that affect cognitive function or acupuncture, massage, application, etc. The clinician will formulate a corresponding treatment plan and make detailed records if the symptoms are severe.

#### 3.6.2. Healthy participants

The healthy participants will not receive any treatment. Matched healthy individuals without cognitive impairment will be determined based on their medical history and cognitive function tests. The general information and cognitive assessment results will be recorded. fMRI images, faecal samples and fasting blood samples of healthy participants will be acquired at enrollment.

### 3.7. Primary outcome

Changes in the Alzheimer’s Disease Assessment Scale-cognitive (ADAS-cog) score, assessed from the baseline to end of treatment, will be used to evaluate the cognitive function of patients. The ADAS-cog is designed as a rating scale for the severity of cognitive dysfunction in people with AD and comprises 12 subscales designed to assess several cognitive domains, including word recall and recognition, naming, instruction, language comprehension and expression, orientation, praxis, attention and other cognitive abilities. The total score is the sum of the scores of all subscales, with higher scores indicating greater cognitive impairment.

### 3.8. Other outcome measures

#### 3.8.1. Changes in clinical symptoms

The following clinical efficacy-related outcomes in patients with aMCI will be used for this study: improvement of cognition, daily living ability, mental behaviour, emotion, and sleep. These outcomes will be measured at baseline and after 12 weeks of the intervention. Each participant will be evaluated by a professional physician unaware of the intervention regimen. Medication combination, compliance, dropout, and elimination cases will be recorded.

The Auditory Verbal Learning Test – Huashan version (AVLT-H) scale will be adopted to evaluate the memory function of patients. It is an objective test proven to be a sensitive measure for diagnosing aMCI (40). The word list consists of 12 two-character Chinese words from three semantic categories (flowers, occupations, and apparel), with four words in each category. The AVLT-H score is allocated as follows: (1) the immediate recall total score representing verbal working memory; (2) the short-term and long-term delayed recall score expressing recall memory; (3) the category-cued recall score; and (4) the recognition score. Considering that delayed recall memory is the first domain to be impaired in individuals with MCI (41), we will measure the changes in long-term delayed recognition scores in this study. The higher the score, the better the memory function.

The Montreal Cognitive Assessment-Basic (MoCA-B) scale will be used to evaluate the cognitive function of patients. The MoCA-B scale is an effective cognitive tool for detecting MCI among older Chinese individuals across all education levels (41). The MoCA-B assesses nine cognitive domains (executive function, language, memory, attention, orientation, calculation, concentration, conceptual thinking, and visual perception) with a total score of 30. The cut-off score for MCI is 16–22 for individuals with mid-level education and 17–24 for those with high-level education. The lower the score, the worse the cognitive function.

The FAQ will be used to evaluate the daily living ability of patients. It is a 10-item measure of difficulties in activities of daily living (42); each item is rated from 0 (no difficulty or independent) to 3 (dependent). The total severity (total sum score from all 10 items, range 0–30) reflects the extent of functional impairment. A total score > 9 indicates impairment of daily living ability; the higher the score, the lower the daily living ability.

The HAMD and HAMA scales will be used to evaluate the emotional status of patients. Anxiety and depression have been identified as risk factors for progression from aMCI to AD dementia (43). Approximately 90% of patients with AD have a variety of psychiatric symptoms, of which depression is the most common type (44, 45). The HAMD scale comprises 17 items that measure somatic and affective symptoms of depression. Each item is scored for severity on a scale of 0–4, with a higher score reflecting higher symptom severity. A total score of 0–7 is generally considered normal; ≥ 7 points indicate probable depression; ≥ 17 points, depression; and ≥ 24 points, severe depression. The HAMA scale comprises 14 items, and each item is rated from 0 (absent) to 4 (severe enough to affect daily life). The total score is the sum of the individual scores of the 14 items. The scoring standards for HAMA are: < 7 points, no anxiety; ≥ 7 points indicate probable anxiety; ≥ 14 points, anxiety; and ≥ 29 points, severe anxiety.

The Mild Behavioral Impairment Checklist (MBI-C) scale will be used to evaluate the mental behaviour of patients. Mild behavioural impairment (MBI) has been proposed as an early manifestation of dementia. The MBI-C is a reliable tool for identifying psychological and behavioural changes in patients with MCI (46). It includes 34 items organised in five domains (decreased motivation, affective dysregulation, impulse dyscontrol, social inappropriateness, and abnormal perception and thought). For each item, a “yes” or “no” question is followed by a severity rating scale of 1 – mild, 2 – moderate, or 3 – severe. MBI-C is especially useful for detecting MBI in people with MCI; the total score is the sum of scores of 34 items, with higher scores indicating the worse MBI.

The Pittsburgh Sleep Quality Index (PSQI) will be used to assess the sleep quality of the participants in the last month before and after the intervention. Sleep disturbances are common in people with MCI and may accelerate MCI progression (47). The PSQI provides a global sleep quality score based on seven components (sleep quality, latency, duration, efficiency, disturbance, use of sleep medication and daytime dysfunction). The sum of scores for these seven components yielded one total score. A global PSQI score > 5 is considered poor sleep quality.

#### 3.8.2. Changes in brain function

fMRI images of all patients will be acquired using a 3.0 T superconducting magnetic resonance scanner (Siemens Medical, Erlangen, Germany) before and after the intervention. All participants will be instructed to keep their eyes closed and bodies aplanatic, and not to think or fall asleep during scanning. The scanning procedure will include a T1 – weighted imaging (T1WI) and BOLD-fMRI. The scanning parameters will be as follows: (1) T1WI: repetition time (TR)/echo time (TE)/T1 = 2000 ms/30 ms/900 ms, field of view (FOV) = 240 mm × 240 mm, voxel size = 0.9 mm × 0.9 mm × 0.9 mm, matrix = 256 × 256, flip angle (FA): 8°, and slice thickness: 0.9 mm; (2) BOLD-fMRI: TR/TE = 2000 ms/30 ms; matrix = 64 × 64, FOV = 240 mm × 240 mm, voxel size = 3.8 mm × 3.8 mm × 4.4 mm, FA = 90°, and slice thickness = 4.4 mm.

#### 3.8.3. Changes in gut microbiota

Faecal samples of all patients will be collected at the beginning and end of the intervention. In the morning, participants will be requested to collect mid-medial faecal samples (at least 2 g) using a disposable stool kit. After the samples are obtained, they will be stored at − 20°C freezers, sent to the laboratory, and placed in the refrigerator at −80°C within 24 h for preservation. We will use a metagenomic sequencing technique to determine the gut microbiota of participants. The DNA of gut microbiota from faecal samples will be extracted using the kit. Subsequently, we will establish a DNA library (paired-end; insert length 350 bp of each sample) according to the manufacturer instructions of Illumina, and conduct high-throughput sequencing using paired-end readings of length 2 × 100 bp. Subsequently, cluster analysis, assembly and annotation of metagenomic data will be conducted. Finally, the annotation, composition, differences, and comparison of species in the intestinal microbiota of patients will be analysed, and the similarities and differences in the diversity and abundance of gut flora will be compared between the AG and WG.

#### 3.8.4. Expression of inflammatory cytokines

Fasting blood samples of all patients will be collected at the beginning and end of the intervention. After obtaining the samples, a serum sample from the participants will be collected using sterile storage tubes; next, we will weigh 500 μL serum sample per tube using a sterilised centrifuge tube, and obtain multiple tubes of each sample for backup. The collected sample will be stored at − 20°C freezers, sent to the laboratory, and placed in the refrigerator at − 80°C within 24 h. Human serum tumor necrosis factor-α (TNF-α), interleukin-1*β* (IL-1*β*), interleukin-4 (IL-4), interleukin-6 (IL-6), interleukin-10 (IL-10), interleukin-18 (IL-18), cyclooxygenase-1 (COX-1), and cyclooxygenase-2 (COX-2) enzyme-linked immunosorbent assay (ELISA) kit will be used for detection.

#### 3.8.5. Others

Drug use, health education completion, acupuncture expectation and treatment satisfaction will be evaluated for patients at the beginning and end of the intervention.

### 3.9. Safety assessments

Physical examinations and vital sign tests are required for all participants. If any adverse events (AEs) occur, a detailed record will be made, the participant will receive appropriate treatment immediately, and the cause of the AEs will be analysed. Most AEs after acupuncture are mild, including the sensation of soreness and distention or localised ecchymoma and bruising that disappear after rest and pressing. Serious AEs will be reported to the ethical committee of the Affiliated Hospital of Chengdu University of TCM in time. When an AE occurs, participants will decide whether or not to quit the trial based on their own will.

### 3.10. Quality control, data management, and monitoring

Before the implementation of this trial, a clinical study manual will be developed, and all investigators will receive specialised training to familiarise them with the clinical study protocol and ensure consistency in the evaluation of efficacy outcomes. Additionally, acupuncture procedures will be stipulated to ensure the accuracy of the acupuncture technique, and acupuncturists will be supervised irregularly. The case report forms will retain all original data of patients and be stored in a secure and restricted access environment. The data will then be entered into a pre-designed, password-protected electronic database by an investigator unaware of the group assignments. Only designated members of the research team will have access to the database. To ensure privacy, all study research documents will be stored in a cabinet in a locked office in Chengdu. The ethical committee of the Affiliated Hospital of Chengdu University of TCM may inspect the study records and monitor the trial.

### 3.11. Statistical analysis

Data analysis will be performed using IBM SPSS version 21.0 (IBM Corp, New York). All data of clinical symptom outcomes will be analysed according to the intention to treat population. The Chi-Square test/Fisher’s Exact test will be used to analyse and count data. Continuous variables as means ± standard deviation or median (± interquartile range), and categorical variables as the number and percentage of participants.

The measurement data includes various scale scores, indicators of brain function, DNA library of gut microbiota, and expression levels of inflammatory cytokines. After exploratory analyses, for variables with normal distribution, independent samples *t*-test will be used for inter-group comparison, and paired samples *t*-test will be used for intra-group comparison. The Mann–Whitney U test will be used for non-normal distributed variables. The correlation analysis between clinical efficacy, gut microbiota, inflammatory cytokines, and brain function will be conducted using multiple linear regression analysis. *p* < 0.05 will be considered the threshold for statistical significance.

## 4. Discussion

We proposed a randomised controlled trial to investigate whether acupuncture can exert therapeutic effects in aMCI. The relationship between clinical efficacy and the gut-brain axis will also be explored.

The gut-brain axis, which is composed of the gut microbiota, central nervous system (CNS), blood–brain barrier (BBB), and various cytokines, has a profound impact on the brain and has multiple effects on memory, mood and behaviour (48). The human gastrointestinal tract is home to diverse microbial community genomes known as the “gut microbiome”. Accumulated evidence indicates that gut microbiota dysbiosis plays a crucial role in the onset and progression of MCI (23). Contrarily, MCI can also cause changes in the diversity and abundance of intestinal microbiota *via* the gut-brain axis (49). Studies have found that the gut microbiota genera in patients with MCI were similar to that in patients with AD but differed from that in healthy controls, and the alteration of microbiomes was correlated with the clinical severity score (50). In gut microbiome dysbiosis, gut microbiota can generate neurotoxic substances like *lipopolysaccharide* (LPS) and *trimethylamine*, which trigger the immune cells residing in the brain and activate the immune system, leading to neuroinflammatory responses and causing heightened gut permeability (51, 52), thereby influencing BBB and brain function. Data suggest that patients with MCI express greater levels of LPS and pro-inflammatory cytokines in the blood compared to healthy participants (53, 54).

Additionally, strong evidence supports that neuroinflammation is key in chronic neurodegenerative disease progression (55), and the occurrence of MCI is closely related to the neuroinflammatory response. Neuroinflammation serves as a broad range of immune responses in the CNS with microglia and astrogliosis as its pathological markers (56), presented by elevated cytokines and inflammatory mediators, causing synaptic disturbances and neuritic dystrophy thus contributing to the initial cognitive decline (57). For example, increased basal production of pro-inflammatory cytokines (IL-1*β*, IL-6, and TNF-α) and decreased production of anti-inflammatory cytokines (IL-4 and IL-10) were observed in senescence-accelerated mouse models (58). TNF-α is a multi-potent, inflammatory cytokine that can induce apoptosis *via* receptor activation (55); TNF-α messenger ribonucleic acid levels are related to the degree of apoptosis in hippocampal neurons, which is closely related to cognition (55). IL-1*β* can upregulate the production of other pro-inflammatory cytokines, prostaglandins, and toxic mediators by initiating a vicious cycle of biochemical pathways (59). Moreover, COX-2 is an inflammatory molecule related to neurodegeneration and associated with synaptic functioning and memory formation, the expression of which is mainly observed in neurons (55). Researchers found that acupuncture reduces LPS, TNF-α and IL-1*β* concentration and regulates BBB disruption in animal models of AD by adjusting the gut microbiota (49). Therefore, the gut-brain axis may be the target for acupuncture treatment of aMCI.

Recently, increasing number of randomized clinical trial (36, 60, 61) have recruited healthy volunteers and determined the pathological features in the population of patients by comparing them with patients. Patients with MCI were reported to have altered levels of peripheral and cerebrospinal fluid inflammatory markers, abnormal cognitive-related brain network function, and distinct gut microbiota composition compared with healthy controls (62–64). In order to discover potential biomarkers associated with aMCI, in this study, healthy participants will be enrolled to assess fMRI, gut microbiota, and inflammatory cytokines, and the differences between patients with aMCI and healthy controls will be identified. The findings will help us to understand the gut-brain axis characteristics of patients with aMCI.

According to the theory of TCM, aMCI belongs to the “amnesia” category, caused by a deficiency of kidney essence and brain marrow. The brain is the marrow sea and the house of the original spirit. The heart stores the spirit and controls mental activities. When the original spirit is damaged, disease occurs. The GV 20 is an acupoint of the governor vessel which runs through the brain and is the most frequently used acupoint in the treatment of MCI (65). GV 24 is located at the anterior part of the frontal lobe and is closely related to memory and thinking. Studies (66, 67) have found that acupuncture at GV24 or GV20 based on the gut-brain axis can relieve gastrointestinal symptoms and regulate the gut microbiome. EX-HN 18 is an extraordinary acupoint for insomnia, associated with cognitive decline in older adults. Treatment of insomnia can help prevent cognitive decline during ageing (68, 69). HT 7 is a specific acupoint of the heart meridian, which can regulate the mind and emotion, research shows that later-life emotional disorder increase the risk of cognitive impairment (70), and the gut microbes are involved in the onset and development of mood disturbance (71). It has been discovered that the gut-brain axis may be the common target of TCM treatment for insomnia and emotional diseases (72, 73). Besides, mindful awareness practice can ameliorate cognitive impairment and modulate the gut microbiome (74). KI 3, an acupoint of the kidney meridian, has the effect of tonifying the kidney and thus nourishing the brain marrow. A Delphi expert consensus survey (75) recommended selecting acupoints on the heart and kidney meridian for cognitive impairment. Thus, the above acupoints were selected to treat aMCI in this study.

The protocol has some limitations. First, the patients and acupuncturists cannot be blinded due to the nature of the intervention. In order to minimise the performance and detection bias, allocation concealment will be conducted in this study, and outcome measures will be taken by an assessor blinded to group allocation and intervention. Second, a fixed acupuncture regimen is designed and will be used for every participant in the trial without syndrome differentiation, which may fail to show the full efficacy of acupuncture. Third, the sample size is relatively small. The potential limitations of this study may impact the results, which may lead to the need for future studies.

This is the first randomised trial to integrate acupuncture into the gut-brain axis in patients with aMCI. Brain function, gut flora, and neuroinflammatory cytokines are indicators of functioning of the gut-brain axis. The differences in the above indicators between patients with aMCI and healthy individuals will be identified to determine gut-brain interaction characteristics in patients with aMCI. Additionally, changes in these indicators between the AG and WG before and after treatment will also be investigated to illustrate the potential mechanism of acupuncture’s therapeutic effects on patients with aMCI. The main advantage of this study is the collection and evaluation of multifaceted data. We hypothesise that acupuncture therapy can improve clinical symptoms in patients with aMCI by regulating the gut-brain axis. If the hypothesis is proven, acupuncture therapy would not only serve as a novel alternative approach for treating aMCI, but also offer new information to further clarify the mechanism of the effects of acupuncture.

## Ethics statement

The local ethical committee of the Affiliated Hospital of Chengdu University of TCM has approved the study protocol (ethical approval number: 2022KL – 041).

## Author contributions

FRL was the study sponsor. FRL, LZ, and ZHY conceived the study. QNB, YWL, and XYZ wrote the first draft of the current protocol, with FRL, ZHY, and LZ providing input to the final draft. ZHY, YQL, QNB, ZQW, FY, XH, YWL, XYZ, MZX, ZHC, JY, WQZ, and KXW were in charge of recruiting participants. ZHY, YWL, YQL, and XYZ provided treatment. ZWW and MSS carried out statistical analysis in the trial. JC and XJH were responsible for the quality control of the trial. All authors contributed to the article and approved the submitted version.

## Funding

This study was financially supported by the Central financial transfer payment to local projects in 2022 of the National Administration of Traditional Chinese Medicine.

## Conflict of interest

The authors declare that the research was conducted in the absence of any commercial or financial relationships that could be construed as a potential conflict of interest.

## Publisher’s note

All claims expressed in this article are solely those of the authors and do not necessarily represent those of their affiliated organizations, or those of the publisher, the editors and the reviewers. Any product that may be evaluated in this article, or claim that may be made by its manufacturer, is not guaranteed or endorsed by the publisher.
